# IgE multiple myeloma: detection and follow-up

**DOI:** 10.1515/almed-2021-0087

**Published:** 2021-12-30

**Authors:** Beatriz Nafría Jiménez, Raquel Oliveros Conejero

**Affiliations:** Department of Clinical Biochemistry, Hospital Universitario Donostia, San Sebastián, Spain; Servicio de Análisis Clínicos, Hospital Universitario Donostia, San Sebastián, Spain

**Keywords:** immunoglobulin E, multiple myeloma, proteinogram

## Abstract

**Objectives:**

We report a new case of immunoglobulin E multiple myeloma (IgE), a very rare isotype that accounts for <0.1% of cases of this monoclonal gammopathy. To ensure the adequate detection, quantification and identification of the monoclonal component, it is crucial that protein assays are performed. We provide some clues related to clinical laboratory results, which will facilitate an adequate management of the disease.

**Case presentation:**

A 45-year-old patient with a five-week history of pain at the level of the elbow, who was diagnosed with IgE-Kappa multiple myeloma based on laboratory, radiological, and bone marrow findings. The patient received induction chemotherapy prior to hematopoietic stem-cell transplantation and is currently on follow-up.

**Conclusions:**

Protein assays performed in the clinical laboratory, including protein electrophoresis and immunofixation, allowed for the detection of an IgE-Kappa monoclonal component prior to the appearance of the typical CRAB symptoms (hypercalcemia, renal involvement, anemia, and bone pain) of multiple myeloma (MM). The detection of IgE-Kappa facilitated early diagnosis and management.

## Introduction

Monoclonal gammopathies embrace a heterogeneous group of diseases characterized by the clonal proliferation of B lymphocytes or immunoglobulin-secreting plasma cells (Ig). These cells secrete Ig molecules both, complete and/or as fractionation products such as free light chains (FLC), among others. They can be detected in serum or urine as a monoclonal component (MC) or paraprotein, also known as protein M. The clinical spectrum is wide, including classical malignancies such as multiple myeloma (MM) or Waldenström macroglobulinemia, clonal paraprotein-related diseases such as light chain amyloidosis, and premalignant plasma cell dyscrasia, called monoclonal gammopathy of undetermined significance (GMSI/MGUS) [1].

More specifically, MM is the second most common hematopoietic malignancy in developed countries, and accounts for 1% of all cases of cancer. As many as 40,000 new cases are diagnosed every year in Europe, 2,200 in Spain. The mean age of patients at diagnosis is 65 years, although it may occur from 45 years of age [2]. MM is classified according to the type of monoclonal heavy chain, as follows: IgG (accounting for 52%), IgA (21%), IgD (2%), IgM (0.5%) and, exceptionally, IgE (<0.1%). Likewise, 16% of cases of MM are FLC-secretors (Kappa or Lambda), although some are non-secretors [3].

The intervention of a laboratory specialist is crucial for diagnosis, as they will perform the detection, quantification and identification of the MC in serum and urine. They will also provide essential data about disease profile, progression and response to treatment. This will enable accurate diagnosis, staging and prognosis, and will help tune-up therapeutic decision-making.

We report a new case of IgE MM, which incidence is so low that only 63 cases have been reported in English [3] since it was first described in 1967 [4].

## Case presentation

We report the case of a 45 year-old man admitted to the Emergency Department of Donostia-San Sebastian University Hospital, Spain, with a 5-week history of severe mechanical pain at the level of the elbow, without further concomitant symptoms. His medical history included arterial hypertension and degenerative changes in the spine and right shoulder, detected the year before. Additionally, the patient reported a 2-cm decrease in height.

Physical examination was normal; however, upon suspicion of right-side epicondylitis, an X-ray study was performed, demonstrating a lytic diaphyseal lesion. In the light of these findings, the patient was hospitalized for further examination (Table 1).

**Table 1:** Laboratory test results at admission.

	Result	Values of reference (VoR)
**(A) Biochemistry**		
Glucose, mg/dL	104	70–110
Creatinine, mg/dL	0.73	0.7–1.2
Urea, mg/dL	49	10–65
Total proteins, g/dL	7.8	6.6–8.7
Calcium, mg/dL	9.6	8.6–10.2
β2-microglobulin, mg/L	2.3	0.8–2.2
Total bilirubin, mg/dL	0.2	0.0–1.1
LDH, U/L	115	135–250
PCR, mg/L	9.8	0–5
Iron, µg/dL	61	59–158
Ferritin, ng/mL	131	30–400
Transferrin, mg/dL	262	200–400
Proteins in 24-h urine, mg/24 h	101	28–141
**(B) Hemogram**		
Erythrocytes, ×10^6^/10^6^/µL	4.1	4.3–5.6
Hemoglobin, g/L	12.1	13–17
Hematocrit, %	38	40–50
MCV, fL	93.4	80–97
MCH, pg	29.7	27–33
MCH, g/dL	31.8	32–36
ADE, %	14.8	11.5–15.6
Platelets, ×10^3^/10^3^/µL	365	140–400
Leukocytes, ×10^3^/10^3^/µL	6.37	3.8–10
Neutrophils, ×10^3^/10^3^/µL (%)	3.09 (48.5)	1.6–7.5 (40–75)
Lymphocytes, ×10^3^/10^3^/µL (%)	2.3 (36.1)	0.9–3.5 (19–48)
Monocytes, ×10^3^/10^3^/µL (%)	0.8 (12.2)	0.2–0.9 (3.5–12)
Eosinophils, ×10^3^/10^3^/µL (%)	0.1 (2.2)	0.0–0.6 (0.5–7.0)
Basophils, ×10^3^/10^3^/µL (%)	0.1 (0.5)	0.0–0.2 (0–1.5)
Peripheral blood morphology	White series: without significant morphological alterations, absence of plasma cells.	
	Red cell series: Rouleaux.	
	Platelet series: high PDW, without significant dysmorphisms.	
**(C) Proteinogram**		
Albumin, g/dL, %	4.5 (58.5)	3.5–4.8 (55.8–66.1)
α1, g/dL, %	0.3 (4.0)	0.1–0.3 (2.9–4.9)
α2, g/dL, %	0.8 (10.7)	0.5–0.9 (7.1–11.8)
β, g/dL, %	0.6 (8.3)	0.6–1.1 (8.4–13.1)
γ, g/dL, %	1.4 (18.5)	0.7–1.6 (11.1–18.8)
CM, g/dL, %	1.33 (17.2)	–
**(D) Inmunoglobulin and light chains in serum**		
IgG, mg/dL	333	700–1,600
IgA, mg/dL	14	70–400
IgM, mg/dL	6	40–230
IgE, kUA/L	4,540,000 (1.1 g/dL)	0.0–114.0
CLL *κ*, mg/L	222.4	3.3–19.4
CLL *λ*, mg/L	1.9	5.7–26.3
K/λ ratio	117.69	0.26–1.65

(A) Serum biochemistry (COBAS c702 Roche^®^, Barcelona, Spain). (B) Hemogram (SYSMEX XN-9100 Roche^®^, Barcelona, Spain) and peripheral blood morphology (Cellavision^®^). (C) Serum proteinogram (Capillarys-2 Sebia^®^, Barcelona, Spain). (D) Quantification of immunoglobulins (COBAS c702 Roche^®^) and light chains in serum (Freelite Assay, The Binding Site^®^, Barcelona, Spain). LDH, lactate dehydrogenase; CRP, C-reactive protein; MCV, mean corpuscular volume in blood; MCH, erythrocyte mean corpuscular hemoglobin; MCH, mean corpuscular hemoglobin; RDW, red cell distribution width; MC, monoclonal component; FLC, free light chains.

Serum laboratory results demonstrated elevated β2-microglobulin levels and mild anemia, along with the presence of Rouleaux in the red cell series (Table 1A and B).

**Figure 1:**
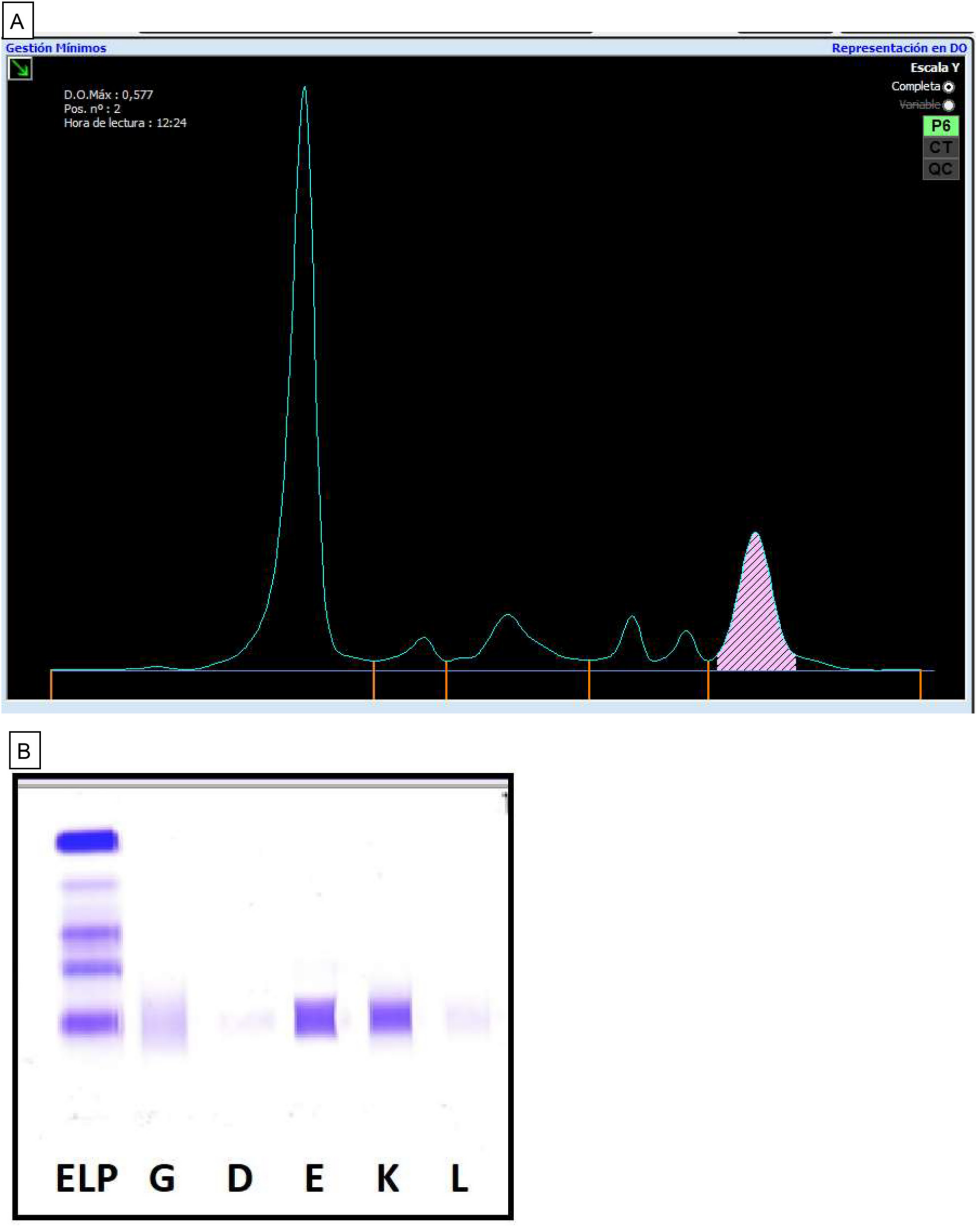
Serum protein test of the patient. (A) Serum proteinogram (Capillarys-2^®^ Sebia) demonstrating a peak that corresponds to the MC in the *γ* fraction. (B) Serum immunofixation (Hydragel IF, Hidrasys Sebia^®^) demonstrating the IgE-Kappa MC (bands corresponding to the regions of anti-igE and anti-CLL Kappa antisera).

Capillary electrophoresis of serum proteins showed a monoclonal peak in the gamma fraction (γ), which was quantified as 1.33 g/dL (Figure 1A). This peak was typified as FLC-Kappa monoclonal component by immunosustraction using anti-IgG, IgA, IgM, and Kappa and Lambda antisera. The result was confirmed and complemented by immunofixation including anti-IgD and anti-IgE antisera. Immunofixation showed a band in the IgE region and also in the Kappa light chain assay, which confirmed the presence of a IgE-Kappa monoclonal proteins (Figure 1B).

With regard to serum immunoglobulin quantification, we observed an elevation in IgE concentrations (ImmunoCAP, Phadia^®^, Barcelona, Spain) and in the Kappa/Lambda light chain ratio in serum, which was 117.69 (Table 1D). 24-h urine analysis did not reveal proteinuria and capillary electrophoresis and immunofixation excluded Bence-Jones MC.

In the light of the results obtained, a bone marrow study was carried out, demonstrating a plasma cell infiltration of 30%, with the totality showing an aberrant immunophenotype by flow cytometry (CD138+/CD38+low/CD19−/CD45+het/CD56++/CD117−/CD27+het/CD81+het). Cytogenetic analysis by *in situ* florescence hybridization (FISH) of purified plasma cells was normal, being IGH-CCND1, t(11;14) (q13;q32) rearrangement the only anomaly detected.

In the light of these findings, and according to the International Myeloma Working Group (IMWG) [1, 5], the patient was diagnosed with IgE-Kappa multiple myeloma, Durie-Salmon stage IIIA, ISS-R:1 [1].

## Discussion

IgE MM is the least frequent type of this monoclonal gammopathy. As a result, knowledge of this disease is based on single reports and small case series [3, 6].

Neoplastic plasma cells and the production of monoclonal Igs damage organs and tissues, resulting in the appearance of the typical clinical manifestations of MM, including hypercalcemia, kidney failure, and hematopoietic alterations with associated anemia and osteolytic bone lesions, which are summarized under the acronym CRAB [7]. These symptoms were also described in the IgE MM cases reported in the literature. Several studies document laboratory results similar to those of other more frequent MM isotypes [8]. However, the case reported here does not meet classical CRAB criteria, since calcium levels in serum and kidney function were normal. Thus, the detection of MC in serum and identification as IgE-Kappa by immunofixation was crucial for establishing the diagnosis, along with other studies including an X-ray and bone marrow study. Therefore, the use of inexpensive assays such as the proteinogram, added to the quantification and identification of monoclonal Ig allows for early diagnosis and helps optimize therapeutic decision-making; as a result, survival outcomes will improve. In agreement with other authors and the IMGW, the screening for MC protocol in the clinical laboratory should include electrophoresis, FLC determination in serum, and immunofixation, which increases sensitivity and facilitates detection [9].

IgE MM distribution by sex and age-at-onset was obtained from the data available for IgE MM, including the case reported here, with a woman-to-man ratio of 20:18, and an age interval of 38–80 years [3, 6].

With regard to first-line treatment in relatively young MM patients (<70 years), we recommend an induction treatment to collect hematopoietic progenitor cells, followed by myeloablative chemotherapy conditioning to eliminate abnormal cells, leave space in the bone marrow for new cells and prevent rejection of new cells in the receptor. Then, autologous peripheral hematopoietic progenitor transplantation (APSCT) is performed [7].

**Figure 2:**
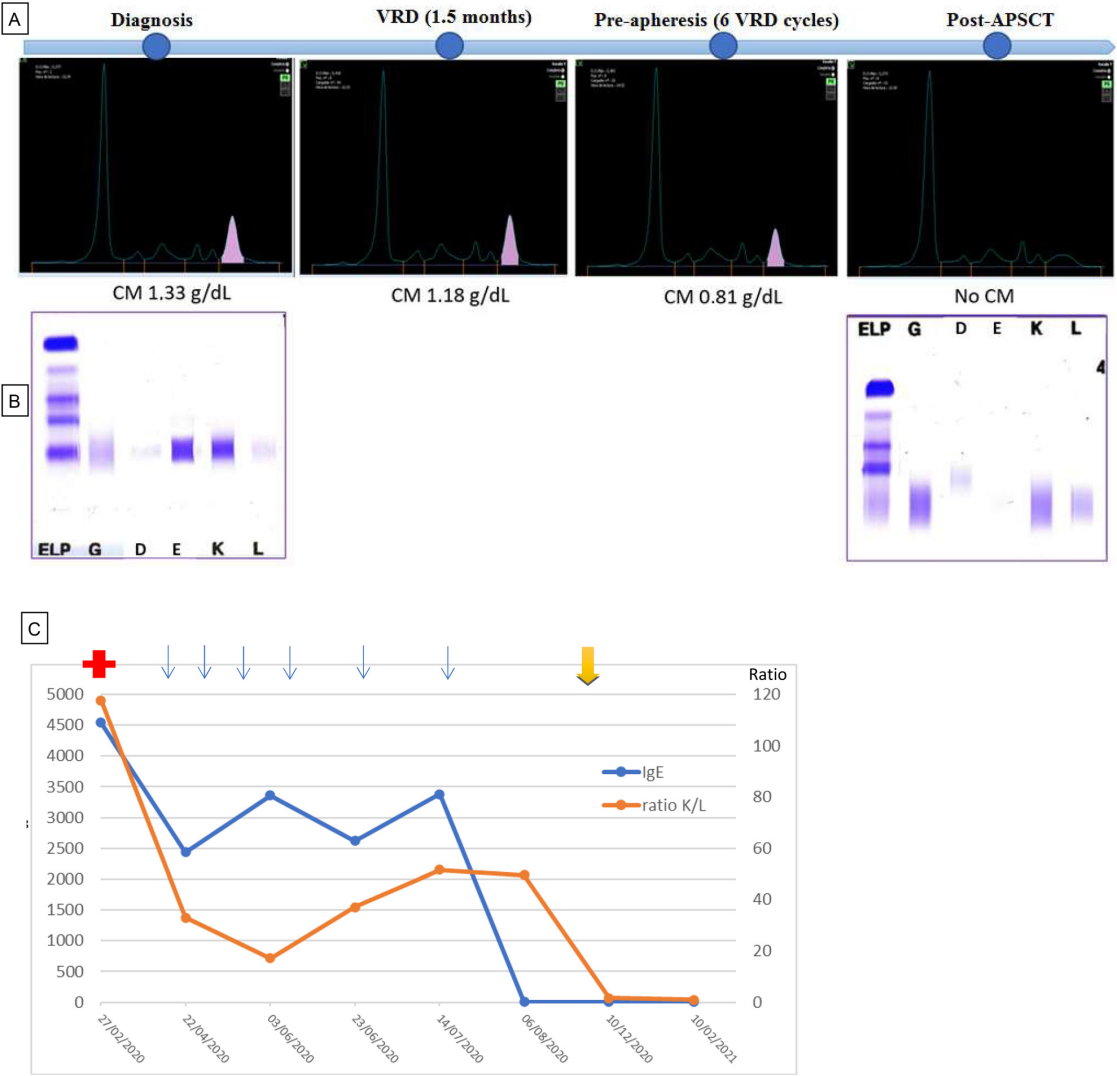
Progress of the protein test of the patient. (A) Progress of the serum proteinogram of the patient: at diagnosis, with the VRD treatment, pre-apheresis and post-APSCT. The monoclonal peak in the *γ* fraction progressively decreases. (B) Serum immunofixation. The IgE-Kappa MC is observed at diagnosis and disappears after transplantation. (C) Progress of serum IgE levels and Kappa/Lambda ration. The initial red cross indicates diagnosis, each blue arrow represents a VRD cycle, whereas the yellow arrow indicates the APSCT. A decrease in serum IgE and CLL-Kappa is observed.

The therapeutic approaches to IgE MM are the same as for more common types of MM, since similar outcomes have been reported [10, 11]. In our case, the patient received an induction regimen of six cycles with the VRD triplet, including Bortezomib (26S proteasome inhibitor), Lenalidomide (immunomodulator) and Dexamethasone (glucocorticoid); and an APSCT. Currently, the patient maintains complete response, with minimal residual disease (persistence of a very small count of malignant cells following treatment), in accordance with IMGW criteria [7]. The patient is on follow-up. Neither the most recent laboratory analysis (one year after diagnosis), nor proteinogram or immunofixation in serum shows the presence of IgE-Kappa MC (Figure 2A and B). Likewise, IgE levels and Kappa/Lambda ratio have progressively decreased during the treatment course (Figure 2C).

The effect of prozone should be, however, considered during serum IgE determination [12]. This limitation is due to the method employed, which is based on the antigen-antibody reaction in a compound balanced for detection. This way, if a patient’s sample shows excess IgE concentrations, anti-IgE antibody saturation occurs, IgE binding develops only partially and the resulting complexes are not formed, which reduces detection. This may yield an incorrect result, which can be prevented with the combined validation of all assays [13]. In our case, IgE concentration at diagnosis was 4,540,000 kUA/L (1.1 g/dL) which is consistent with the concentrations estimated in the proteinogram (1.33 g/dL).

With regard to cytogenetic abnormalities (FISH), only the IGH-CCND1 t(11;14)(q13;q32) rearrangement was detected, without any further chromosomal alterations. In this sense, the t(11;14) translocation is a distinctive marker of IgE MM, IgM and non-secreting MM, since this translocation is significantly more frequent in rare subtypes of MM [14].

With regards to prognosis, the literature postulates a shorter mean survival (12.5 months) even after APSCT, and a higher rate of disease progress to plasma cell leukemia [8]. However, the data available was obtained when novel immunomodulators and proteasome inhibitors had not yet been introduced in clinical practice. The most recent evidence shows that clinical outcomes have improved notably with the use of these new agents [10]. Nevertheless, delayed diagnosis of rare MM may result in a more aggressive clinical course. Thus, the theory that IgE MM has poorer prognosis is currently a subject of debate. Indeed, there is a case report of a patient who survived 20 years and eventually died at 77 from chronic comorbidities [3].

In summary, IgE MM is a rare type of myeloma which clinical characteristics and therapeutic approach are similar to those of other isotypes. Further studies are needed for general conclusions about this condition to be drawn, and our results should be interpreted with caution. Anyway, this case report contributes to the current body of knowledge about IgE MC detection.

## Learning points

– Although immunoglobulin E multiple myeloma is a rare condition, this isotype should be considered when a monoclonal component is initially identified.
– The proper use of protein assays facilitates diagnosis and staging and is crucial for a correct evaluation and prognosis.
– For the detection of a monoclonal component, electrophoretic proteinogram is recommended. Likewise, the gold-standard method for MC identification is immunofixation with specific anti-IgE antisera.
– The effect of prozone is a widely-known laboratory phenomenon that may yield misleading quantitative results, resulting in falsely normal results. This is especially relevant for IgE MM, which may remain undiagnosed.
– Establishing protocols for the detection, isotype identification and follow-up of monoclonal gammapathies based on electrophoresis, immunofixation, and immunoglobulin and free light chain quantification will help us better understand the impact of early detection and treatment on clinical outcomes.
